# Wide Complex Tachycardia as a Rare Pointer of Intra-aortic Balloon Pump Migration Into the Left Ventricle: A Case Report and Literature Review

**DOI:** 10.7759/cureus.59789

**Published:** 2024-05-07

**Authors:** Kyrillos Girgis, Desmond Aroke, Danielle Retcho, Grettel Gonzalez Garcia, Sammir S Dekowski, Rafail Beshai, Jessica Celenza-Salvatore, Fazal Ali

**Affiliations:** 1 Internal Medicine, Newark Beth Israel Medical Center, Newark, USA; 2 Cardiovascular Disease, Virtua Health, Camden, USA; 3 Internal Medicine, Jefferson Health, Stratford, USA; 4 Cardiology, Newark Beth Israel Medical Center, Newark, USA

**Keywords:** intra-aortic balloon pump, complications, ventricular tachycardia, left ventricular unloading, cardiogenic shock

## Abstract

Intra-aortic balloon pumps (IABPs) are used to assist with left ventricular (LV) unloading in patients with cardiogenic shock (CS). There are different mechanical devices that can be used in CS, of which the IABP represents the simplest, the easiest to insert and remove, and the most cost-effective. Compared to traditional femoral IABPs, axillary IABPs allow patients to remain ambulatory. This is especially beneficial in patients awaiting heart transplants. Our case presents a patient with CS, where axillary IABP was used to unload the LV. However, our patient developed ventricular arrhythmia secondary to IABP migration to the LV.

## Introduction

Hypoperfusion caused by acute exacerbation of heart failure with reduced ejection fraction (HFrEF) is associated with high short-term mortality. There are different mechanical devices that can improve the survival rate, of which the intra-aortic balloon pump (IABP) represents the simplest, the easiest to insert and remove, and the most cost-effective [1]. Complications of IABP usually include limb ischemia, visceral ischemia, stroke, deep or superficial vein thrombosis, infection, IABP leak, poor inflation, or poor augmentation [2].

Ventricular tachycardia is defined as a wide complex tachyarrhythmia with QRS greater than 120 milliseconds and a heart rate greater than 100 beats per minute. The most common cause of ventricular tachycardia is ischemic heart disease. Other causes include adult and congenital structural heart disease, infiltrative cardiomyopathy, illicit drugs such as cocaine or methamphetamine, digitalis toxicity, and electrolyte imbalances such as hypokalemia, hypocalcemia, and hypomagnesemia [3].

Herein, we present a case of a cardiogenic shock (CS) secondary to HFrEF exacerbation treated with IABP. However, the patient developed a very rare complication of ventricular tachycardia due to IABP dislocation.

## Case presentation

A 69-year-old male with a past medical history significant for HFrEF on high dose home Primacor, mitral regurgitation status post bio-prosthetic mitral valve replacement, non-ischemic cardiomyopathy, status post automatic implantable cardioverter-defibrillator (AICD), atrial fibrillation on warfarin, and hypothyroidism presented for a routine right heart catheterization for evaluation of heart transplant candidacy. The patient reported fatigue and shortness of breath. He stated that he was able to walk about one block which had not changed recently. He reported using two pillows for comfort. He denied paroxysmal nocturnal dyspnea, chest pain, palpitations, dizziness, AICD shocks, or syncope.

The routine right heart catheterization was done which demonstrated elevated filling pressures and low cardiac output/cardiac index on high-dose Primacor. The pulmonary artery catheter was left in place for further monitoring and the patient was admitted for further management.

**Figure 1:**
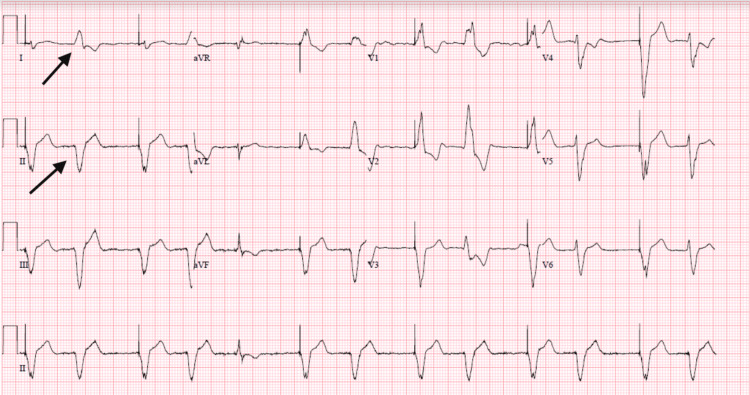
An electrocardiogram showing ventricular paced rhythm (black arrows)

**Figure 2:**
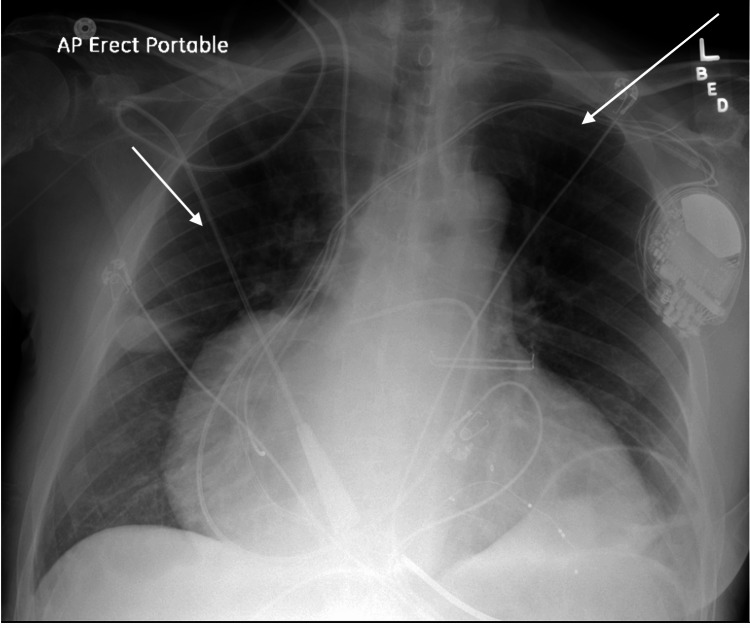
A chest X-ray showing mild pulmonary vascular congestion (white arrows)

On admission, vital signs showed blood pressure of 130/80 mmHg, heart rate of 71 beats per minute (BPM), respiratory rate of 18 breaths per minute, and oral temperature of 97.7 °F. Physical examination did not show significant abnormalities apart from a distended internal jugular vein. An electrocardiogram (EKG) showed pacemaker rhythm with wide QRS complexes and left axis deviation (Figure 1). Labs were significant for an international normalized ratio (INR) of 2.4, and brain natriuretic peptide of 700. A chest X-ray showed mild pulmonary vascular congestion with no focal consolidation or pleural effusion noted (Figure 2). A trans-thoracic echocardiogram (TTE) was done, which showed severe left ventricular (LV) dilation with an LV ejection fraction of 15-20%, severe right ventricular hypokinesis with moderate right ventricular dilation, severe bi-atrial enlargement, bioprosthetic mitral valve was well seated with no regurgitation, and moderate to severe tricuspid regurgitation was noted. 

The patient was started on intravenous (IV) Lasix and dobutamine was added for inotropic support. The home dose of levothyroxine 175 mcg was continued. Warfarin was held, and a heparin drip was started when INR became less than two. 

A few days after admission, the patient's blood pressure dropped to 85/55 mmHg despite being on dobutamine. The patient was informed about the importance of inserting IABP, and he signed the consent. A left axillary IABP was inserted in the Cath lab using the Seldinger technique without any complications.

About 12 hours after the insertion of the axillary IABP, the patient's heart rate became 150 BPM. At that time, the patient was awake, alert, and oriented. He denied chest pain and shortness of breath but endorsed a feeling of palpitations. Telemetry showed sustained ventricular tachycardia. The patient received 100 mg of IV push lidocaine, a 150 mg IV amiodarone bolus followed by continuous infusion, along with 2 gm of IV magnesium. 

**Figure 3:**
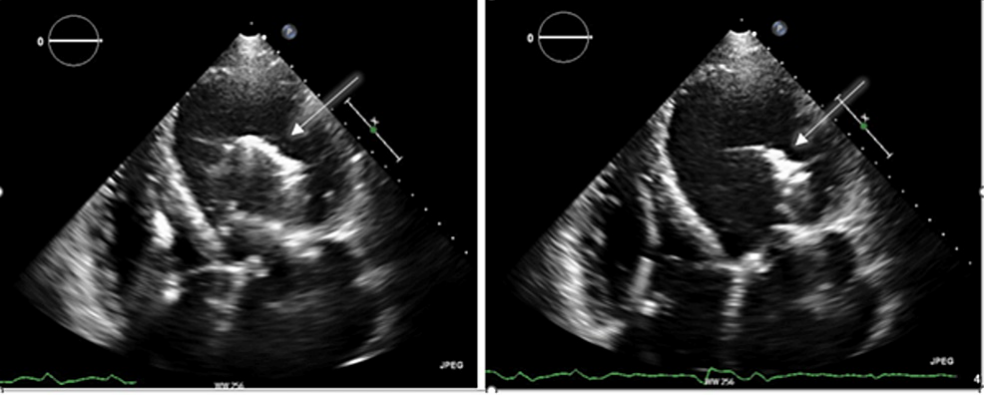
A four-chamber apical view TTE showing an echo dense mass (white arrow) representing the IABP in the LV TTE: trans-thoracic echocardiogram; IABP: intra-aortic balloon pump; LV: left ventricular

**Figure 4:**
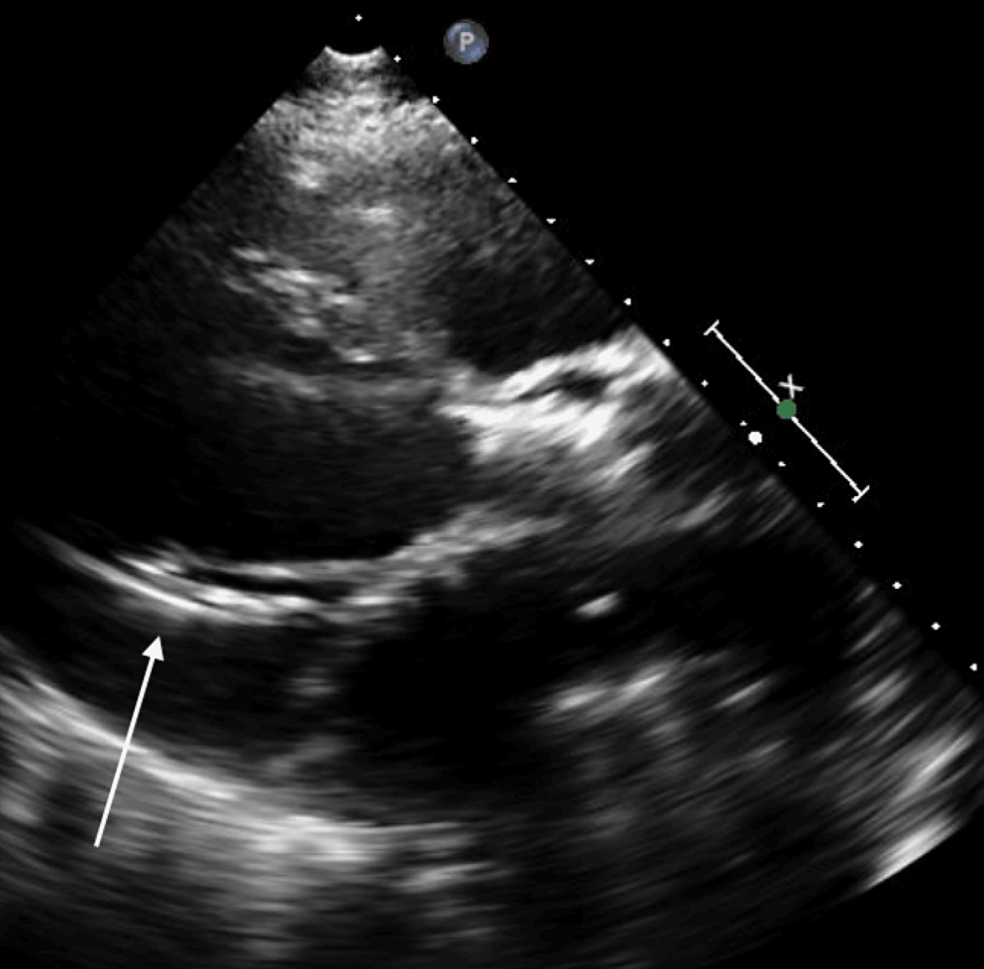
A long parasternal view TTE showing an echo dense mass (white arrow) representing the IABP in the LV TTE: trans-thoracic echocardiogram; IABP: intra-aortic balloon pump; LV: left ventricular

**Figure 5:**
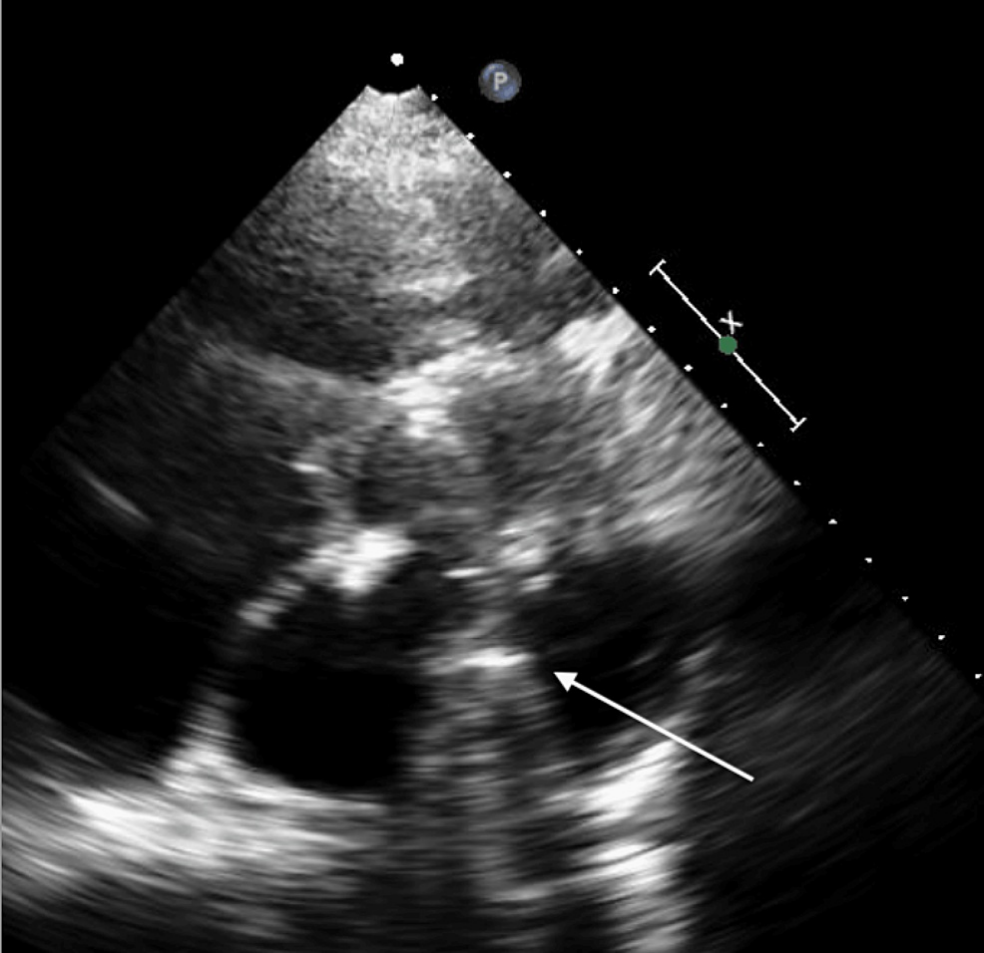
A short parasternal view TTE showing an echo dense mass (white arrow) representing the IABP in the LV TTE: trans-thoracic echocardiogram; IABP: intra-aortic balloon pump; LV: left ventricular

A STAT bedside ECHO (echocardiogram) was performed due to abnormal IABP waveforms (Figures 3-5). This showed IABP migration into the LV cavity. The IABP was immediately stopped and the patient was rushed to the Cath lab. The axillary IABP was removed and replaced with a femoral IABP. The patient's course thereafter was uneventful, and he was successfully transplanted. Although the IABP was secured at two locations and all precautions were taken during ambulation, the device had migrated spontaneously.

## Discussion

CS is a potentially fatal illness related to cardiac pump malfunction that causes end-organ hypoperfusion and hypoxia. CS is defined by a low cardiac index of less than 1.8 L/min/m^2^ without support or 2.0-2.2 L/min/m^2^ with support, systolic blood pressure of 90 mmHg or less, and symptoms of systemic hypo-perfusion including increased lactic acid without hypovolemia [4]. The increasing number of patients with congestive heart failure who are resistant to guideline-directed medical therapy has resulted in the development of a number of mechanical circulatory support (MCS) devices that can be used to restore systemic perfusion, allowing cardiac recovery in the short term, and/or as a bridge to transplantation in refractory heart failure [5]. There are different types of MCS including IABP, Impella (Abiomed, Danvers, MA, USA) non-percutaneous ventricular assist devices, and extracorporeal membrane oxygenation (ECMO) devices [1].

Among the various MCS types, IABP is considered the simplest, most affordable, and easiest to implant and remove in the cardiac catheterization laboratory by an interventional cardiologist [1]. IABP was invented by Dr. Kantrowitz and was used for the first time in 1968 [6]. The IABP is used to augment the diastolic filling of the coronaries and unload the left ventricle. The mechanism by which the IABP works is the diastolic inflation of the balloon synchronously with aortic valve closure leads to an augmentation of the diastolic pressure that improves the perfusion of the coronaries and reduces the myocardial oxygen demand. The systolic deflation of the balloon creates a suction force that helps the movement of blood from the left ventricle to the aorta, which explains the left ventricle unloading [7].

Acute exacerbation of heart failure refractory to medical treatment as in our case is considered among the most common indications of IABP in the USA. Other indications include adjunctive in high-risk percutaneous coronary intervention (PCI), acute myocardial infarction (MI) causing CS, low cardiac output after coronary artery bypass graft (CABG), and as a bridge to definitive treatment in patients with refractory heart failure [1]. Contraindications to IABP include moderate to severe aortic regurgitation, aortic aneurysm, aortic dissection, uncontrolled infection, or uncontrolled bleeding diathesis [1]. 

Complications of IABP include vascular complications (most common) such as visceral ischemia, limb ischemia leading to amputations, and deep and superficial vein thrombosis. Other complications include infection, IABP leak, poor inflation, or poor augmentation [2]. Our case presents a very rare complication of ventricular arrhythmia secondary to IABP dislocation into the left ventricle. 

The IABP used to be inserted via the transfemoral approach. However, percutaneous axillary IABP has evolved and is now preferred over transfemoral IABPs due to their improved mobility and less debilitation. The most common complication of trans axillary IABPs is pump migration commonly to the descending aortic and upwards to the subclavian. To the best of our knowledge, only one case of LV migration has been reported in a patient with concomitant veno-arterial extracorporeal membrane device support [8]. Our case is unique as our patient had no other intra-aortic devices and the IABP has migrated against the flow into the LV.

## Conclusions

It remains unclear if there were any pre-disposing anatomic factors to consider. When considering axillary IABP to promote ambulation; clinicians should remain vigilant about spontaneous IABP migration post-ambulation. Device position should be confirmed daily and after any episode of ambulation.
